# Literacies and the Development of Social, Critical, and Creative Thought in Textbook Activities for Primary Education in Social Sciences and the Spanish Language

**DOI:** 10.3389/fpsyg.2019.02572

**Published:** 2019-11-13

**Authors:** Delfín Ortega-Sánchez, Esther Sanz de la Cal, Jaime Ibáñez Quintana

**Affiliations:** Department of Specific Didactics, Faculty of Education, University of Burgos, Burgos, Spain

**Keywords:** reading conception, literacies, social thinking, textbook activities, primary education

## Abstract

The skills of thinking, reading conceptions and reading practice (literacy levels) found in textbook activities for the sixth year of Primary Education in Social Sciences and Spanish Language in Spain are analyzed in this paper. A mixed methodology is used to triangulate the data, integrating the critical analysis of discourse and two types of statistical analysis: descriptive (frequencies and percentages) and inferential (χ^2^, ANOVA, and the Mann-Whitney *U* Test). The results inform us of both the permanence and the strengthening of the design of an activity oriented toward the development of traditional conceptions of linguistic-cognitive reading. In the framework of education for global citizenship, the conclusion is the need for student reading practices that begin with the principles of critical literacy directed at the acquisition of social, critical, and creative thinking skills.

## Introduction

An analysis of the conceptions of reading promoted by the school textbook has to start, in the first place, with the decodification of its languages and narrative grammars. This “reading for decodification” finds itself subject to subjectivizing and adaptation, at all times determined by the hermeneutic perspective of subjects and groups, and the dependence and temporality of their category as a cultural production, reflection of thoughts, ideologies and generator of collective memories.

Cassany (2006) proposed three approaches or levels of textual reading: the *linguistic conception*, the *psycholinguistic conception*, and the *sociocultural conception*; the latter necessary in literacies that are capable of questioning social discourse and of revealing ideologies, silences and exclusions. The first of them assumes meaning as the objective reality, inherent to the text, neutralizing all interpretative possibilities and directing the practice of reading toward the repetition of contents that are presumably pre-established and immutable. From cognitive psychology, the second conception contemplates the practice of reading as an interactive and integrated system between the reader and the text, where the revelation of the textual meaning lies in the inferential reconstruction and the meaningful readjustment of the message of the author in the cognitive schemes of the reader. Finally, the sociocultural perspective approaches the textual semantic field, and its understanding in terms of the social and cultural referents of the community to which the receiver belongs, understanding the practice of reading as a social practice. This perspective affirms the origin of the preconceptions and the cognitive schemes of the reader that are of an eminently social and cultural nature, involving knowledge of the discursive genre and the sociocultural context of the textbooks under consideration. In this way, the practices of literacy are turned into important constructors of hegemonic social and cultural models of power.

It is here where we conceptualize the term critical literacy as the knowledge, capabilities and attitudes, and values derived from the generalized, historic, individual and social use of the written code (Cassany, 2006). Its curricular integration in the school textbook is a priority in the deconstruction of the present, past, and future social narrative, always discursive and, therefore, intentional and directed at forming beliefs, representations and social practices. It may be affirmed that critical literacy is the practice of analyzing texts from a critical point of view, with the purpose of adopting a proactive position toward controversial topics in the context of global citizenship based on human rights.

Critical literacy responds to an idea of committed and active social practice that emerges in a specific culture and context for social transformation (Santisteban et al., 2016). This concept is not, therefore, attached to a definite set of methodological rules or to the ultimate end of acquiring specific cognitive capabilities or learning standards, but on the adoption of critical (Cassany, 2006), deconstructive and participative positions (Patel and Bean, 2007) toward the social narrative (Lankshear and McLaren, 1993; Van Dijk, 1993, 1999, 2003; McDaniel, 2004; Ross, 2004; Knobel, 2007; Mulcahy, 2008; Wallowitz, 2008). From this perspective, “critical literacy should direct itself toward action, should imply decision/making, commitment and intervention in social problems (Santisteban et al., 2016).

We agree with Lewison et al. (2002) in considering the question of information (*sociopolitical texts*), intentional or ideological analysis of the information (*disrupting the commonplace*), the textual analysis of different points of view (*considering multiple viewpoints*), and a will to transform (*taking action*), as the fundamental underlying structure of literacy criticism. These dimensions transcend the traditional reading-writing capabilities of (literacy comprehension and inferential comprehension of texts) to achieve critical competencies oriented toward understanding and reflection on textural intentionality, and the actions in response to contemporary social problems (social-cultural and critical understanding) (Alderson, 2000; Livingstone et al., 2008; Quintero, 2009; Llusà and Santisteban, 2017). Accordingly,

Students (…) learn to take a stance toward news, reports and text, in order to analyze their nature as facts or opinions, their veracity, trustworthiness, intentionality and omissions, and thereby to position themselves actively in a social context (Tosar and Santisteban, 2016, p. 675).

The Spanish curriculum of Spanish Language and Literature for Primary Education (6–12 years old) defines the reading skills as the main access ways to all areas, and insists on the need for the students to be able to reconstruct explicit and implicit ideas in diverse texts, in order to build their own critical and creative thinking. In a complementary and integrated way, the Social Science curriculum for this educational stage, in particular, for the last year (year six) prescribes the proposal of activities that allows the students to perform the social reality and actively take part in it, from the elaboration of value judgments autonomously and the development of critical and creative skills for the effective resolution of problems (DECRETO 26/2016, 2016). In this sense, the acquisition of competencies linked to critical literacy has special interest for the critical reading of the social reality, a curricular guideline only included in these two subjects. To achieve this objective, students, therefore, have to know how to read the lines (literal understanding), read between the lines (inferential understanding) and read behind the lines (understanding of the point of view or ideology- critical understanding-) (Ortega-Sánchez and Pagès, 2017). These readings skills are adapted, adequately, to the cognitive characteristics of the students of the last year of Primary Education (12 years), when logical thinking about abstract concepts emerges (stage of formal operations) and when social perspective is acquired, learning to manage and assimilate, at the same time, both one’s own point of view and the Other (Peñacoba et al., 2006).

The present study attempts, through the assumptions of critical literacy and the critical conception of reading as a social practice, to analyze the underlying conceptions and practices of reading in the design of activities proposed in Primary Education textbooks for the teaching of Social Sciences and the Spanish language. On the understanding that literacy is the *way of using* reading and writing in the framework of a specific social mission (Zavala, 2008), the investigation attempts to study the specific contribution of school textbooks to democratic reading education, the development of receptive critical competence, and the promotion of critical and creative thought in students on the last course of this educational stage. On the basis of these objectives, four research hypotheses are proposed in this study (H_1_):

H_1(01)_: the epistemological nature of the curricular subjects (Social Sciences and Spanish Language) influences the promotion or limitation of reading conceptions aimed at the critical textual understanding for social transformation.

H_1(02)_: school publishing stamps influence the educational promotion of reading practices related to critical literacy.

H_1(1)_: the reading conceptions and thinking skills promoted in the curricular activities are different in the subjects: Social Sciences and Spanish Language.

H_1(2)_: the reading conceptions and thinking skills promoted in the curricular activities are different depending on the commercial publisher.

## Method

### Sample

The selected sample is composed of a total of 4477 activities, included in three of the most extensive educational publishing houses in Spain: Santillana, Anaya and SM. With a view to the evaluation of the skills of social, critical and creative thought promoted in student textbooks, the selection of the units of analysis (curricular subjects) was in line with intentional and exploratory criteria, prioritizing Spanish language and Social Sciences textbooks, because of their epistemological nature, from the last year of Primary Education (Table 1).

**TABLE 1:** Distribution of activities by subject and editorial.

	**Spanish language**	**Social sciences**	**Total**
Santillana	1299	565	1864
	41.7%	41.4%	41.6%
Anaya	820	383	1203
	26.3%	28.1%	26.9%
SM	994	416	1410
	31.9%	30.5%	31.5%
Total	3113	1364	4477
	100.0%	100.0%	100.0%

### Instrument

The instrument in use consisted of a page of data records, taken from a system of aprioristic categories that have shaped the conception of literacy, the level of reading, the promotion of cognitive skills, and the type of activity linked to the texts under study. The categorical assignment of the activities began with the methodological assumptions of the critical analysis of discourse and the “learning of comprehension” for critical and democratic censorship (Londoño, 2015).

From the principles of critical theory and the cognitive-constructivist perspective, the definition of these categories of analysis has adapted the three approaches to the reading of Jurado (2008) (literal reading, inferential reading, and intertextual reading) and the conceptions of Cassany (2006) (linguistic conception, psycholinguistic conception, and sociocultural/socio-political conception), as well as their potential correspondence with the levels of cognitive complexity of the revised taxonomy of Bloom (Anderson and Krathwohl, 2001).

Of the six skills of thinking proposed in the taxonomic revision of Bloom, the capabilities of analysis and application are included in another two cognitive capabilities of a higher order: the skills applied in the processes of evaluation and creation. We undertake to resolve problems in new situations as one of their basic working principles; and the analysis of problems that relate to the understanding of the textual meaning, in so far as their deconstruction requires a review of the previous cognitive schemes and their mobilization by the student.

Likewise, in the activity of a critical comprehension of the social reality, and assuming that reading and writing are not ends in themselves, but forms of achieving wider social objectives and cultural practices (Zavala, 2008), we adopted the sociopolitical conception of critical literacy of Cassany and Castellà (2010). In relation with this conception, when the variables of analysis permitted the identification of two sub-levels of reading in the same procedural design, the activity was codified and analyzed at more than one level of progress (Table 2).

**TABLE 2:** Progressive levels of cognitive-reading.

**Category**	**Dimension**			**Variable**	**Descriptor**
**Conception of reading**	**Level of reading**	**Cognitive capability**	**C**	**Associated activity**	
Linguistic conception	Literal literacy	Lower order	N1	Recall	Literal decodification of the textual meaning.
Psycholinguistic conception	Inferential literacy	Medium order	N2	Understand	Inferential understanding of the textual meaning.
Sociocultural conception/Sociopolitical transformation	Critical literacy	Higher order	N3	Evaluate	Critical textual understanding for social transformation.
			N4	Create	

*C: Code.*

The analysis variable that allows identifying the reading levels is included in the column named “variable.” The columns “dimension” and “category” describe, develop and conceptualize the indicators of the “linked activity” based, as indicated, on the revision of Bloom’s Taxonomy. An example of the data casting and analysis applied can be found at the following link: http://cort.as/-IhAy The reliability of this categorization that owed a lot to the independent collaboration of a fourth researcher achieved a degree of agreement of 98% in the coding of the 4477 activities under consideration.

### Design and Procedure

The investigation was developed with the method of mixed concurrent triangulation design (Creswell and Plano, 2007), seeking to correlate, compare, and contrast the data obtained from following both qualitative and quantitative methodologies in a simultaneous manner. The investigation is approached, on the one hand, through an interpretative-descriptive, qualitative and applied focus, as it attempts to offer criteria with which to evaluate the identifiable levels of literacy in the activities of the educational textbooks. The application of content analysis techniques is, on the other hand, combined with an applied descriptive and inferential statistical analysis of the data.

The data gathering process and the scope of the conclusions depended on an inductive procedure, a characteristic of qualitative investigations, on the basis of systematic observation of the reality under analysis. Nonetheless, the study also followed the procedures that formed part of non-experimental *ex post facto*.

### Data Analysis

The units to be recorded were identified by an alphanumerical code, in accordance with the subject matter and the publishing house. Each unit was sorted under one of the three aprioristic approaches or categories under consideration, their level of reading, cognitive capabilities, and the associated activities. Finally, their frequency of appearance in the unit of analysis was quantified and the data were categorized.

ATLAS.ti software was used for data analysis and processing, due to its organizational capability and information management of a qualitative nature. The researchers applied the categories system described in Table 2 to proceed to the emptying of data. The information was emptied in Excel sheets in order to facilitate the recording of each level of progression.

The numerical data were processed with SPSS v.24 software. A descriptive statistical analysis was performed on the basis of frequencies and percentages of total and inferential results, through χ^2^, and comparative hypothesis testing. The atypical distribution of the data was established following the application of the Kologorov/Smirnov test, so we therefore used the non-parametric Mann-Whitney *U* and Kruskal-Wallis tests for independent samples.

## Results

### Conception of Linguistic Reading: Literal Literacy

In accordance with the results of the screened data, 23.4% of the 2694 activities assigned to the first level of progress corresponded to the subject of Social Sciences and, very notably, 76.6% to the Spanish Language (Table 3).

**TABLE 3:** Levels of cognitive-reading progress (N1 – literal literacy).

	**Spanish language**		**Social sciences**	
Santillana	*f.*	615	*f.*	255
	%	70.7	%	29.3
Anaya	*f.*	647	*f.*	195
	%	76.8	%	23.2
SM	*f.*	802	*f.*	180
	%	81.7	%	18.3
Total	*f.*	2064	*f.*	630
	%	76.6	%	23.4

*Frequencies and percentages.*

The designs of these activities require textual reproduction and the retrieval of stable and objective textual meanings that are found in the narrative itself. The activities developed on the basis of this conception give no consideration to decision-making for social development, interpretation, declarations concerning the intentionality of explicit ideas and values in the textual material, and the questioning of its discursive nature, with the aim of developing the critical conscience of the students.

The level of reading of these activities are specified in the development of literal literacies through the acquisition of cognitive skills of a lower order, with no need for the prior knowledge of the students and the social, cultural and historic context in which its contents are situated: “Copy the diagram into your notebook and complete it with the names of the monarchs and regents that are missing” (Martín et al., 2015, p. 70). With this approach, reading activities of different sorts are frequently proposed, which then involve text retrieval activities: “Write a summary of *The legend of rice* (Arenillas et al., 2015, p. 133).”

### Conception of Psycholinguistic Reading: Inferential Literacy

Equally significant are the proposals designed in terms of psycholinguistic conceptions (1406 activities), including activities oriented toward the understanding of social and historic contents (37.8%), and grammatical and literary contents (62.2%) (Table 4).

**TABLE 4:** Levels of cognitive-reading progress (N2 – inferential literacy).

	**Spanish language**		**Social sciences**	
Santillana	*f.*	583	*f.*	176
	%	76.8	%	23.2
Anaya	*f.*	144	*f.*	159
	%	47.5	%	52.5
SM	*f.*	147	*f.*	197
	%	42.7	%	57.3
Total	*f.*	874	*f.*	532
	%	62.2	%	37.8

*Frequencies and percentages.*

Beyond their understanding, these reading and text management practices do not appear to transcend intervention in local or global social and cultural environments, personal interaction and, in consequence the sociopolitical use of the contents that are covered. In effect, the dominion of this functional literacy is presented here as a self-sufficient phenomenon that excludes room for social action and social usage of the test. It cannot, therefore, be affirmed that the design of these activities conceives the text and its management as a truly contextualized social practice.

Its reading level, expressed in the development of inferential literacies, is defined in the framework of the development of cognitive capabilities of medium order, on the basis of the interpretation the textual meaning, its explanation, and the incorporation of a more immediate context: “What means of transport and communication do you use on a daily basis? Did they exist in the Modern Age?” (Martín et al., 2015, p. 77).

In the fulfilment of these procedures, the students need to start with their own previous knowledge to achieve the objectives of the activity (Bello et al., 2015, p. 201):

*The Story of a Shipwrecked Sailor*. Change the following text into a literary narration in the first person: ‘The shipwrecked sailor felt a pain in his right knee. He saw that the wound was deep and that it was already dry thanks to the seawater’.

### Socio/Cultural Conception of Reading: Literacy Criticism

Sociocultural and sociopolitical teaching units were found in 277 activities in the third sub-level of progress (evaluation), and in 100 activities at the fourth sub-level (creation). Among them, 48.4% (evaluation) and 68% (creation) of the records corresponded to the Social Sciences, and 51.6% (evaluation) and 32% (creation) to Spanish Language (Tables 5, 6).

**TABLE 5:** Levels of cognitive-reading progress (N3 – literacy criticism).

	**Spanish language**		**Social sciences**	
Santillana	*f.*	75	*f.*	71
	%	51.4	%	48.6
Anaya	*f.*	28	*f.*	26
	%	51.9	%	48.1
SM	*f.*	40	*f.*	37
	%	51.9	%	48.1
Total	*f.*	143	*f.*	134
	%	51.6	%	48.4

*Frequencies and percentages.*

**TABLE 6:** Levels of cognitive-reading progress (N4 – literacy criticism).

	**Spanish language**		**Social sciences**	
Santillana	*f.*	26	*f.*	63
	%	29.2	%	70.8
Anaya	*f.*	1	*f.*	3
	%	25.0	%	75.0
SM	*f.*	5	*f.*	2
	%	71.4	%	28.6
Total	*f.*	32	*f*.	68
	%	32.0	%	68.0

*Frequencies and percentages.*

In the area of relevant social problems, or indeed socially alive questions, and the encouragement of social thinking at school, these activities are directed toward action, commitment and social intervention; they attempt to surpass educational proposals, centered on the progressive acquisition of certain standardized cognitive skills of critical thought, to achieve the necessary critical frame-of-mind in the promotion of social transformation. Among other procedures, the student is asked to give an opinion on the meaning of the text at hand, through the mobilization of prior knowledge and the acceptance of social roles: “Investigate why there are beaches that have no blue flag and, from among them all, develop a plan of action to manage it. What could you do?” (Benítez et al., 2015, p. 63).

Along these lines, the design of activities within this approach connects with the social and cultural environment of the students for their critical comprehension: Write a paragraph explaining your opinion on this sentence: “No culture is higher or lower than another, they are only different” (Arenillas et al., 2015, p. 68).

Likewise, it requests the transformation of textual meaning into significant knowledge (Bellón et al., 2015, p. 99), through the adoption of a social commitment and a transformational approach among the students: “Explain how you would feel if children were obliged to work. How would it change your life? Why do you think that it is important that children attend school?” (Calzado et al., 2015, p. 171).

### Hypothesis Tests

The χ^2^ statistic of independence confirms the existence of the relation between the variables Publisher and level of reading (χ^2^_(6, 4477)_ = 292.461, *p* < 0.001), and subject matter and level of reading (χ^2^_(3, 4477)_ = 208.266, *p* < 0.001) (H_1(01, 02)_).

It can be concluded that both the publishing house logo and the type of subject influenced the reading conceptions proposed in the designs of the activities. This relation between variables was supported by the results of the Mann-Whitney U test, noting significant differences between the reading conceptions and the two subjects (Social Sciences and Spanish Language) under study (*U* = 1651571, *p* < 0.001).

These results confirmed H_1(1)_, according to which the reading levels differ in the subjects of Social Sciences and the Spanish Language. These differences are visible in the assignment of the majority of Spanish Language activities to traditional (literal and inferential) reading levels, as against the assignment of the activities proposed for the subject matter of Social Sciences to critical reading levels (Table 7).

**TABLE 7:** Contingency table for the variables subject matter and reading conception.

			**Reading conception**				**Total**
			**N1**	**N2**	**N3**	**N4**	
Subject matter	Spanish language	*f.*	2064	874	143	32	3113
		%	76.6	62.2	51.6	32.0	69.5
	Social sciences	*f*.	630	532	134	68	1364
		%	23.4	37.8	48.4	68.0	30.5
Total		*f*.	2694	1406	277	100	4477
		%	100.0	100.0	100.0	100.0	100.0

Finally, with the intention of corroborating the existence of significant differences between reading levels and the three publishing houses under study, we applied the Kruskal-Wallis ANOVA test of independent groups. The results confirmed that the reading levels differed in accordance with the publishing house associated with the activities under analysis (χ^2^_(2)_ = 255.616, *p* < 0.001).

These results lend support to H_1(2)_ according to which the reading levels and, in consequence, the associated thinking skills will differ in accordance with the publishing house (Table 8).

**TABLE 8:** Contingency table for the variables publishing house and reading conception.

			**Reading conception**				**Total**
			**N1**	**N2**	**N3**	**N4**	
Publisher	Santillana	*f*.	870	759	146	89	1864
		%	32.3%	54.0%	52.7%	89.0%	41.6%
	Anaya	*f*.	842	303	54	4	1203
		%	31.3%	21.6%	19.5%	4.0%	26.9%
	SM	*f*.	982	344	77	7	1410
		%	36.5%	24.5%	27.8%	7.0%	31.5%
Total		*f*.	2694	1406	277	100	4477
		%	100.0%	100.0%	100.0%	100.0%	100.0%

It is confirmed that the representations of the socio-cultural/socio-political conception were the least frequent among all the publishing houses. Within these limited frequencies, the publishing house Santillana stands out, in a positive light, with 52.7% (N3) and 89% (N4) of the procedural designs oriented toward social action.

## Discussion and Conclusion

As shown by the most recent investigations into reading practices in school textbooks (Zárate Pérez, 2010, 2015), we have also confirmed the continuance of linguistic and psycholinguistic focuses in the design of the activities proposed for curricular development in Primary Education in Spain. The predominance of activities of either a cognitive style or of inferential/psycholinguistic reading is confirmed in the school textbooks under analysis; mainly in those used to teach the subject Spanish Language. These results reinforce the dominant tendency of technical curricular focuses in the didactic treatment of the contents at this educational stage. The sociocultural focuses of critical comprehension activities linked to problems of social relevance and current social issues are, in consequence, a minority. They require evaluation, application and connection of the information with social and cultural environment of the student, the questioning of the texts that are proposed, decision/taking, assuming stances and the proposal of alternatives and/or solutions.

In line with the results from classroom studies in Primary and Secondary education in Spain (Santisteban et al., 2016; Tosar, 2017), the absence of any development of competencies in critical literacy is evidence of the continuation of teaching and learning processes centered above all on the acquisition of linguistic and cognitive skills. In accordance with the results of this investigation, these skills are unrelated to social action, democratic participation (Obenchain and Pennington, 2015), democratic education for global citizenship (Davies, 2006; Soares and Wood, 2010), and the analysis of relations between language and power (Knoblauch and Brannon, 1993; Mulcahy, 2008; Luke, 2012).

Despite the special relevance of activities specifically related to critical literacy at the end of the Primary Education stage, the results that were obtained report the absence of the correspondence between the curricular aims of the subjects of Spanish Language and Literature and Social Science and its effective development in textbooks with greatest editorial dissemination. There is no doubt regarding the need to provide students with adequate tools to interpret and to question, through the critical analysis of texts, the broad diversity of multimodal discourses that model social reality. The use of resources of critical literacy in the classroom would in that sense have to surpass the literal and semantic decodification of unidirectional readings, with the purpose of helping children to ask questions on the basis of their own experiences, to discuss possible alternatives (Lankshear and McLaren, 1993; Fisher, 2008) and with the purpose of assuming critical roles capable of identifying textual intentionality, social inequalities and invisibility (Bishop, 2014).

With the aim of approaching an increasingly global and plural environment, from a critical perspective, and assuming that critical reading is a curricular objective, teacher training would have to implement specific programs that might contemplate using the resources of critical literacy (Ortega-Sánchez, 2017) in the framework of education for global citizenship. These resources would have to center on arguments, debate and participation in problems of social relevance, offering future students the necessary tools to learn to think (Wolk, 2003), and to identify the presence or absence of social issues (Ogle et al., 2007), identities and perspectives in the discourses with the capacity to influence their thought and actions.

## Data Availability Statement

The datasets for this manuscript are not publicly available because the data sets are not definitive and are in the process of being extended. Requests to access the datasets should be directed to dosanchez@ubu.es.

## Author Contributions

DO-S: conceptualization, methodology, formal analysis, writing-original draft preparation, review, and editing, visualization, supervision, and project administration. DO-S and JQ: software. DO-S, EC, and JQ: investigation, resources, and data curation.

## Conflict of Interest

The authors declare that the research was conducted in the absence of any commercial or financial relationships that could be construed as a potential conflict of interest.
